# Differences in social preference between the sexes during ontogeny drive segregation in a precocial species

**DOI:** 10.1007/s00265-017-2332-2

**Published:** 2017-06-20

**Authors:** Mark A. Whiteside, Jayden O. van Horik, Ellis J. G. Langley, Christine E. Beardsworth, Philippa R. Laker, Joah R. Madden

**Affiliations:** 1 Centre for Research in Animal Behaviour, Psychology, University of Exeter, Exeter, EX4 4QG UK; 2 Game and Wildlife Conservation Trust, Burgate Manor, Fordingbridge, Hampshire, SP6 1EF UK

**Keywords:** Aggression, Body size dimorphism, Group living, Ontogeny, Social preference, Sexual segregation

## Abstract

**Abstract:**

Hypotheses for why animals sexually segregate typically rely on adult traits, such as differences in sexual roles causing differential habitat preferences, or size dimorphism inducing differences in diet or behaviour. However, segregation can occur in juveniles before such roles or size dimorphism is well established. In young humans, leading hypotheses suggest that (1) sexes differ in their activity and the synchronisation of behaviour causes segregation and (2) sexes separate in order to learn and maximise future reproductive roles. We reared pheasants, *Phasianus colchicus*, from hatching in the absence of adults in a controlled environment. Females aggregated with their own sex from hatching, whereas males initially exhibited random association, but segregation became pronounced with age. The increase in segregation corresponded to an increase in sexual size dimorphism. By standardising habitat availability and diet and by removing predation risk, we could disregard the Predation Risk and the Forage Selection Hypotheses operating at this age. Activity budgets did not differ between the sexes, providing no support for the Behavioural Synchrony or the Activity Budget Hypotheses. Both sexes preferentially approached groups of unfamiliar, same-sex birds in binary choice tests, providing support for the Social Preference Hypothesis. Females may segregate to avoid male aggression. Sexual segregation may become established early in development, especially in precocial species, such as pheasants. A clear understanding of ontogenetic factors is essential to further our understanding of adult assortment patterns. Assortment by sex may not be inherent, but rather emerge as a consequence of social interactions early in life.

**Significance statement:**

Hypotheses pertaining to the force driving sexual segregation typically rely on adult traits, such as size dimorphism or differences in sexual roles. However, in some species, animals segregate as juveniles, so that most hypotheses previously invoked to explain sexual segregation in adults are irrelevant. We reared pheasants, *Phasianus colchicus*, from hatching and monitored multiple aspects of the chicks’ life history in an effort to determine what causes sexual segregation. Females aggregate with their own sex from hatching, whereas males initially have a more random association, but segregation becomes pronounced as both sexes got older, coinciding with greater sexual dimorphism. We controlled for influences of predation risk and dietary/habitat choice and found that activity budgets did not differ between the sexes. Instead, we found that both sexes preferred their own sex when presented with a binary choice, providing evidence that social preference could drive sexual segregation in pheasants.

## Introduction

In most species, segregation, whereby males and females live separated by time and/or space, is only observed when the sexes are old enough to be sexually active (e.g. mouflon, *Ovis gmelini* (Cransac et al. 1998); red deer, *Cervus elaphus* (Alves et al. 2013); red kangaroo, *Macropus rufus* (MacFarlane and Coulson 2005); and northern giant petrels, *Macronectes halli* (González-Solís et al. 2000)). Consequently, hypotheses to explain why segregation occurs typically refer to adult traits, such as differences in reproductive roles, or are related to body size dimorphism, or are a combination of both (Ruckstuhl and Neuhaus 2005). Three main hypotheses are proposed. The Predation Risk Hypothesis states that sexes differ in habitat choice to promote reproductive success (Main and Coblentz 1996; Ruckstuhl and Neuhaus 2002). Males maximise growth by choosing habitats that have higher-quality forage but potentially higher predation risk (Clutton-Brock et al. 1982; Prins 1989). Adult females with dependent young may try to protect them by choosing areas of low predation risk but of lesser quality forage (Corti and Shackleton 2002). The Forage Selection Hypothesis suggests that allometric differences between sexes result in differing morphological characteristics, such as bite size and digestive efficiencies, causing differences in nutrient requirements (Demment 1982; Main and Coblentz 1996; Barboza and Bowyer 2000). Individuals, usually females, with smaller and less efficient digestive systems require diets of higher quality and therefore choose richer habitats (Demment and Van Soest 1985; Barboza and Bowyer 2001). Finally, the Activity Budget Hypothesis suggests that segregation occurs when differences in body size between the sexes causes differences in behaviour, and segregation occurs when members of one sex exhibit the same behaviours simultaneously at different times to members of the opposite sex (Conradt 1998; Ruckstuhl 1998; Ruckstuhl and Neuhaus 2000, 2002).

However, sexual segregation is not restricted to adulthood (Bon et al. 2001), and therefore, hypotheses reliant on adult traits may not apply in these situations. Spider monkeys, *Ateles geoffroyi*, and cowbirds, *Molothrus ater*, segregate as juveniles (Kohn et al. 2011; Rodrigues 2014). Segregation within the natal group suggests that ontogenetic social factors could be important (Gaudin et al. 2015). Understanding why it occurs prior to adulthood may provide insight into how influential social factors are in the development of sexual segregation.

Sexual segregation is commonly reported in young humans. Children as young as 3 years old are reluctant to interact with opposite-sex peers (Serbin et al. 1979), manifesting into segregation when given free choice during unsupervised free time by the age of 7 years (Pellegrini et al. 2002), continuing into adolescence (Brown et al. 1986; Legault and Strayer 1991). Two hypotheses have been suggested to explain segregation during ontogeny in humans. The first, the Energetic/Behavioural Synchrony Hypothesis, is similar to the Activity Budget Hypothesis but does not require sexual size dimorphism. The Energetic/Behavioural Synchrony Hypothesis states that the demands of future reproductive roles, mediated by adult body size, causes males and females to perform separate behaviours in an effort to optimise growth. Human males should be more active than females in order to develop muscles involved with intra-sexual contests (Eaton and Enns 1986; Campbell and Eaton 1999), and consequently, males spending more time developing muscles, perhaps in association with other males, causes segregation. The second, the Social Roles Hypothesis, similar to the Social Factor Hypothesis (Main et al. 1996), states that males and females invest in behaviours that prepare them for adult reproductive roles. Males will practice competitive and dominance roles, in the form of play, and so may seek out male contest partners to practice with (Pellegrini and Smith 1998). In adult western grey kangaroos, *Macropus fuliginosus*, social affinity in males, serving to maximise competitive intra-sexual interactions, drives social segregation (MacFarlane and Coulson 2009). Females will either invest in the learning of maternal roles (e.g. in humans (Saltz et al. 1977) and in chimpanzees, where young females learn to handle infants (Pusey 1990)) and so seek other (older) females to associate with, or use indirect aggression in efforts to form alliances (Campbell and Eaton 1999) and so seek out other females to ally with. Segregation can also occur when females find aggressive play encounters with males unpleasant and hence avoid them (Harper and Sanders 1975; Pellegrini 1992; Smuts 1995).

Although social factors may drive the development of sexual segregation (Villaret and Bon 1995; Bon and Campan 1996), the link between the two is rarely studied in animals (Ruckstuhl 2007). Differences between the sexes in social preferences and behavioural patterns during ontogeny have been suggested to cause sexual segregation in adult ungulates, with the preferences observed in adults arising from preferences developed during early life (Villaret and Bon 1995; Bon and Campan 1996; Cransac et al. 1998). However, this is rarely formally tested. Ontogenetic differences in the motivation to be around same-sex conspecifics have been suggested as a reason for why Soay sheep, *Ovis aries*, and merino sheep prefer their own sex in a choice test (Pérez-Barbería et al. 2005; Michelena et al. 2006); however, these sheep were only tested as adults. Tests on young mouflon sheep reveal that females show a closer affinity to their mothers than males, which could cause social segregation (Gaudin et al. 2015). The reason for the lack of research in this area is threefold. Firstly, many studies are conducted on wild animals. In these systems, it is difficult to tease apart individual hypotheses because this requires the assessment of size dimorphism, attribution of predation risk, measurement of habitat quality and quantifying food availability (Ruckstuhl 2007). Secondly, current study systems typically have few offspring; for instance, multiple births are rare in ungulate species, e.g. big horn sheep, *Ovis canadensis californiana* (Eccles and Shackleton 1979), and Soay sheep (Clutton-Brock et al. 1992). This reduces the number of likely interactions and the opportunities for the young to develop preferences early in life. A lack of segregation in mouflon as yearlings is believed to be due to a lack of numbers in a cohort (Ruckstuhl 1999). Finally, many of the frequently studied species (typically ungulates) have altricial young. Being highly reliant on parents affects the scope for interactions, restricting opportunities to interact with peers. Here, we may see sex bias in parental influence and dispersal; for instance, female mouflon sheep spend more time with their mother than males do (Guilhem et al. 2006). Male chamois, *Rupicapra rupicapra*, show a stronger tendency to disperse earlier than females (Loison et al. 2008). The practicalities of current study systems mean that we may be underestimating the impact that ontogenetic factors have on sexual segregation. A system is therefore required which would allow peers to interact with each other from a young age, with little input from parents, in an environment that can be controlled. Precocial systems with large cohort sizes in which individuals can be reared in captivity may illuminate these relationships.

Pheasants, *Phasianus colchicus*, have many characteristics of the sexually segregating ungulates, but the system allows for the study of sexual segregation during ontogeny. Adult pheasants are highly sexually dimorphic in body size (Wittzell 1991) and in gut morphology (MAW et al. unpubl. data), and crucially, they segregate outside the mating season (Hill and Ridley 1987; Hill and Robertson 1988), prior to reaching maturation (MAW et al. unpubl. data). Pheasants produce a large number of precocial offspring, with average brood sizes of 10.6 (Dumke and Pils 1979). This allows for individuals to interact with siblings and peers and may facilitate the development of sexual segregation from a young age. Critically, pheasants exhibit visible sex differences in morphology from hatching (Woehler and Gates 1970), with males having a small wattle flap under their eye. Dimorphism progresses to include plumage differences, initially noticeable at around 6–7 weeks, and increases over subsequent weeks, such that birds can be sexed by visual inspection by humans with increasing accuracy. We therefore predict that pheasants may sexually segregate during early development.

Pheasants can be reared in captivity from hatching where they can be individually marked, which allows the assessment of patterns of assortment. It is not known when males significantly differ from females in body size, which is crucial for a number of hypotheses. Pheasants can be weighed at regular intervals which allows for assessment of size dimorphism. Finally, they can be reared both in the absence of parents and under highly controlled conditions, allowing many of the hypotheses addressing sexual segregation to be either explicitly tested or discounted.

Sexual dimorphism could still influence sexual segregation, so we tried to address all possible hypotheses in the study (see Table 1 for a summary of predictions). The Predation Risk Hypothesis requires a study species that is sexually dimorphic in body size and that suffers from high predation risk (Main and Coblentz 1990; Miquelle et al. 1992). Measuring assortment prior to reaching maturation removes the influence that caring for young could have on assortment; however, males may still opt for a riskier strategy if there is a benefit for their growth. To control for the influence of predation risk, chicks can be reared in a secure location that is surrounded by wire mesh and electric fence. However, some aspects of predator risk is innate (Rubolini et al. 2015), so in addition, birds can be reared in a homogenous environment which provides no opportunity for habitat choice based on risk of predation. The Forage Selection Hypothesis requires an environment enriched with food items to allow sex-specific preferences to develop due to size differences in gut morphology. Manipulations of the rearing environment, in terms of diet provision, can help tease apart the effects of diet choice on segregation. Firstly, pheasant chicks, which are naturally dietary generalists (Hill 1985), can be reared with a homogenous diet, such as chick crumb, provided in excess and in standardised locations, removing opportunities for diet choice. Alternatively, chicks can be reared with access to a diverse diet, and then an individual’s dietary preferences can be tested in isolation. The Energetic/Behavioural Synchrony Hypothesis requires that behaviour, in particular, foraging and resting, differs between the sexes. The Activity Budget Hypothesis only predicts behavioural differences between sexes during a period when they are dimorphic in body size. In captivity, food provision can be standardised and be in excess, meaning that differences in foraging behaviour are due to a more efficient foraging technique, which can be assayed in pheasants (see Whiteside et al. 2015). The Social Roles Hypothesis requires that birds prefer partners of the same sex in binary choice tests and that males, in efforts to enforce dominance or to improve future reproductive success, are typically more aggressive than females. Pheasant chicks exhibit clear aggressive acts from an early age which include pecking and chasing conspecifics (Butler and Davis 2010), which may drive preferences. Placing pheasant chicks into a binary choice test allows testing of preferences under controlled conditions (see Madden and Whiteside 2013).

**Table 1:** Hypotheses and predictions to explain sexual segregation during ontogeny

Hypothesis	Size dimorphism	Predictions
Predation Risk Hypothesis	Yes	No segregation should occur when monomorphic in body size.No segregation should occur when reared in a homogenous environment, free from habitat differences and without the risk of predation.
Forage Selection Hypothesis	Yes	No segregation should occur when housed in a homogenous environment, free from habitat differences and without access to diverse food.
Energetic Behavioural Synchrony	No	Foraging or resting behaviour should differ between the sexes throughout development.Consistency in foraging behaviour between the sexes could be due to a more efficient foraging strategy.
Activity Budget Hypothesis	Yes	Foraging or resting behaviour should differ between the sexes during periods when they are dimorphic in body size only.Consistency in foraging behaviour between the sexes could be due to a more efficient foraging strategy.
Social Roles Hypothesis	No	Sexes choose to be with the same sex when presented with a binary choice.Males may be more aggressive in an effort to assert dominance or increase future reproductive success.

We reared pheasants, a precocial species, under conditions that controlled for habitat and food availability, reproductive status and predation risk. We determined patterns of social preferences in a semi-natural environment from when they are 1 day old, for 8 weeks. We then assessed when pheasants became sexually dimorphic in body size. Finally, we determined what could influence the patterns of social preferences by assessing behaviour, social preference, dietary choice and foraging efficiency during periods when pheasants were either monomorphic or dimorphic in body size. This allowed us to establish if sexual segregation is an inevitable emergent consequence and a function of behavioural synchrony, due to body size dimorphism driving dietary choice, or due to active choice as indicated by clear preferences in binary choice tests of social partners.

## Methods

### Housing

In June 2010 and May 2012, we purchased 300 1-day-old chicks in each year from a commercial supplier, sexed them using the presence of a wattle as a cue (Woehler and Gates 1970) and randomly allocated them to one of 10 replicated rearing houses in groups of 30 with a 50:50 sex ratio, where they remained for 8 weeks. In both 2015 and 2016, we used 200 1-day-old chicks and randomly allocated them to one of four replicated rearing houses in groups of 50 for 10 weeks. For the first 2 weeks, the birds were confined to enclosures (2010/12, 1.3 m × 1.3 m; 2015/16, 2 m × 2 m) in a heated house. After 2 weeks, the birds had access to an open grass run (2010/12, 1.3 m × 6.8 m; 2015/16, 4 m × 12 m) as well as the house. All birds were supplied with age-specific commercial chick crumb at standardised feed stations ad lib and in excess. In 2015 and 2016, chicks were fed with mealworms and mixed seed and fruit, in addition to the chick crumbs. The houses and the grass runs were surrounded by wire mesh and an electrified fence.

### Do pheasants show a preference to associate with their own sex within a semi-natural environment, and does this change with age?

In 2010, 67 males and 67 females (for the distribution of sample size with week, see Table 2) were visually observed for a maximum of 10 min using a continuous focal follow combined with an instantaneous point sampling, with 30-s intervals. At each interval, we recorded the behaviour of the bird (see *Is segregation a by-product of behavioural synchrony*?) and the sex of its nearest neighbour. A neighbour was defined as an individual positioned within five body lengths of the focal bird. Each bird was observed only once as a focal individual during the study. We used a Generalised Linear Mixed Model (GLMM) with a binomial distribution to assess if males and females differed in their association with their own sex and age (Table 3).

**Table 2:** The number of female and male pheasants observed using a continuous focal follow methods during each week of the study

Week	Male sample size	Female sample size
1	9	9
2	15	15
3	17	17
4	10	10
6	9	9
7	9	9
8	8	8

**Table 3:** The distribution, response variables, explanatory variables and random factors for all GLMMs used in the study

Question	Distribution	Response	Explanatory factors	Random factors
Do pheasants show a preference to associate with their own sex within a semi-natural environment, and does this change with age?	Binomial	Sex of nearest neighbour	Sex of focal; degree of dimorphism	House
Do sexes differ in mass at day 1?	Normal	Mass	Sex of focal	House
Do sexes differ in mass at day 18?	Normal	Mass	Sex of focal	House
Do sexes differ in mass at day 28 and day 56?	Normal	Mass	Sex of focal; age	Bird ID; house
Do sexes differ in their dietary diversity?	Poisson	Number of food items chosen	Sex of focal	House
Do sexes differ in their foraging and vigilance behaviour?	Binomial	Foraging/vigilance	Sex of focal; degree of dimorphism	House
Do sexes differ in their aggression?	Binomial	Aggression towards	Sex of receiver (same/opposite)Sex of focal; degree of dimorphism	Bird ID; house
Do sexes show a preference for a particular sex?	Normal	Time spent with (ArcSinSQRT)	Sex of choice (same/opposite); sex of focal; degree of dimorphism	Bird ID

### When do sexes differ in body size during development?

In 2010, we recorded the mass of a subset of birds at day 1 (*n* = 60), a different subset of birds at day 18 (*n* = 50) and from all birds at days 28 and 55 (*n* = 294). For birds weighed at day 1 and at day 18, we used separate GLMMs to assess if males differ from females in their mass (Table 3). The same birds were weighed at days 28 and day 55; therefore, we used another GLMM to determine if the mass of the birds differed with the sex and the age of the bird (Table 3).

### Do sexes differ in diet selection?

In 2015, we presented 148 (67 females, 81 males) 10-week-old (sexually size dimorphic) chicks with a dietary choice task. The birds voluntarily walked into a test chamber (750 mm × 750 mm) that was connected to the rearing house via a sliding door and were presented with a box (120 mm × 400 mm) containing 10 wells (diameter = 20 mm, depth = 15 mm). All birds were habituated to both the test chamber and the apparatus prior to testing (see van Horik and Madden 2016), allowing for the bird to be tested in isolation. Each well contained a different food item (pumpkin seeds, red dog biscuits, red chick crumb, maize, sunflower seeds, green dog biscuits, raisins, mealworms, standard chick crumb, wheat). Birds were free to choose from all wells. We recorded the order a food item was chosen and the total number of different food items consumed. A bird was released after all wells were chosen or when it had not interacted with a food item for 3 min. We used a GLMM with a Poisson distribution to assess if sexes differed in their dietary diversity, the number of different food items chosen during the task (Table 3). To determine if sexes differed in which food items they preferred during the trial, we used a chi-squared test to investigate if the percentage of the female population that chose a certain food item in their first three choices differed from that of the male population.

### Do sexes differ in the time spent foraging or resting?

At each instantaneous point sample (see “Do pheasants show a preference to associate with their own sex within a semi-natural environment, and does this change with age?” section), we recorded the behaviour of the focal bird. All behaviours were mutually exclusive. Specifically, foraging included eating, drinking and searching for food, identified as walking with the pheasant’s head and neck directed towards the ground, and resting was determined as either standing or lying with eyes closed. We used GLMMs with a binomial distribution to assess if sexes differed in their foraging and resting likelihoods with age (Table 3).

### Does foraging efficiency differ between the sexes?

In 2012, we presented 117 (56 females, 61 males) 4-week-old chicks, during a period when they are sexually dimorphic in body size, with a food handling test, the ‘cricket challenge’ (see Whiteside et al. 2015). In an arena (1.3 m × 1.3 m), we placed a cricket (*Gryllus assimilus*), a novel insect that could be eaten, that was tethered on a 20-cm line and concealed behind a barrier. Into the arena, we placed two randomly selected pheasants. The pheasants in 2012 were not shaped or habituated to humans or the testing environment and would not forage when tested singly; therefore, to reduce the effects of stress, we used pairs of birds for each test. We allowed a 5-min habituation period, after which we removed the barrier to expose the cricket. Both birds were observed during the test. Time of detection of the cricket was recorded for both birds. Pheasants, like any avian species with laterally placed eyes, will first turn their head sideways to inspect food (Bischof 1988; Hodos 1993), so we used this behaviour as a measure of first detection. We then measured the time it took for one of the birds to eat the cricket. We calculated the time it took for the bird to catch the prey by subtracting the time of first detection, for the bird that eventually completed the task, from the time of consumption, and used a Wilcoxon signed-rank test to explore the differences in handling time between sexes. No birds were used in subsequent tests as a companion or a focal.

One potential problem with the cricket challenge was that the cricket moved differently on every occasion, and this may have influenced the speed that it was caught. In addition, the companion bird could affect the behaviour of the focal. Therefore, in 2015, we presented 186 (84 females, 102 male) 10-week-old chicks with the ‘roboworm’, an adaptation of the ‘cricket challenge’, which allowed us to standardise the movement of the prey item. To remove the effects that the companion birds could have on the focal, all birds were previously habituated to both the testing chamber and to being tested in isolation (see van Horik and Madden 2016). A dead mealworm was attached to an arm of fine gauge wire (150 mm) which in turn was attached to the second hand of a crystal-modulated electronic clock. The arm moved in a circle, horizontal to the floor, with a sporadic pattern controlled by overriding the crystal pulses and replacing these with pulses governed by an Arduino physical computing interface. The pattern of pulses was determined randomly such that the worm moved in a series of jerky, sporadic arcs, but the pattern of pulses was identical for each bird. Another mealworm, termed the baseline worm, was placed at the front edge of the apparatus to direct the bird towards the apparatus and standardise their approach to the roboworm. All birds were tested individually. We started timing when the birds pecked the baseline worm, therefore controlling differences in propensity to approach a novel food source, and stopped timing when the bird pecked the roboworm. A Wilcoxon signed-rank test was used to ask if the sexes differed in their handling time.

### Does aggression differ between the sexes?

During the continuous focal follow (see “Do pheasants show a preference to associate with their own sex within a semi-natural environment, and does this change with age?” section), we recorded all aggressive interactions exhibited by the focal bird. These interactions included feather pecking, chasing, fighting and aggressive pecks to vulnerable parts of the body such as the head. For each interaction, we recorded the sex of the receiver. We used a GLMM with a Poisson distribution to assess if sexes differ in (1) the amount of aggression they inflict on others, and (2) the amount of aggression they inflict towards a particular sex, during periods that the birds were sexually monomorphic and dimorphic in body size (Table 3).

### Do sexes show a preference for a particular sex?

**Fig. 1:**
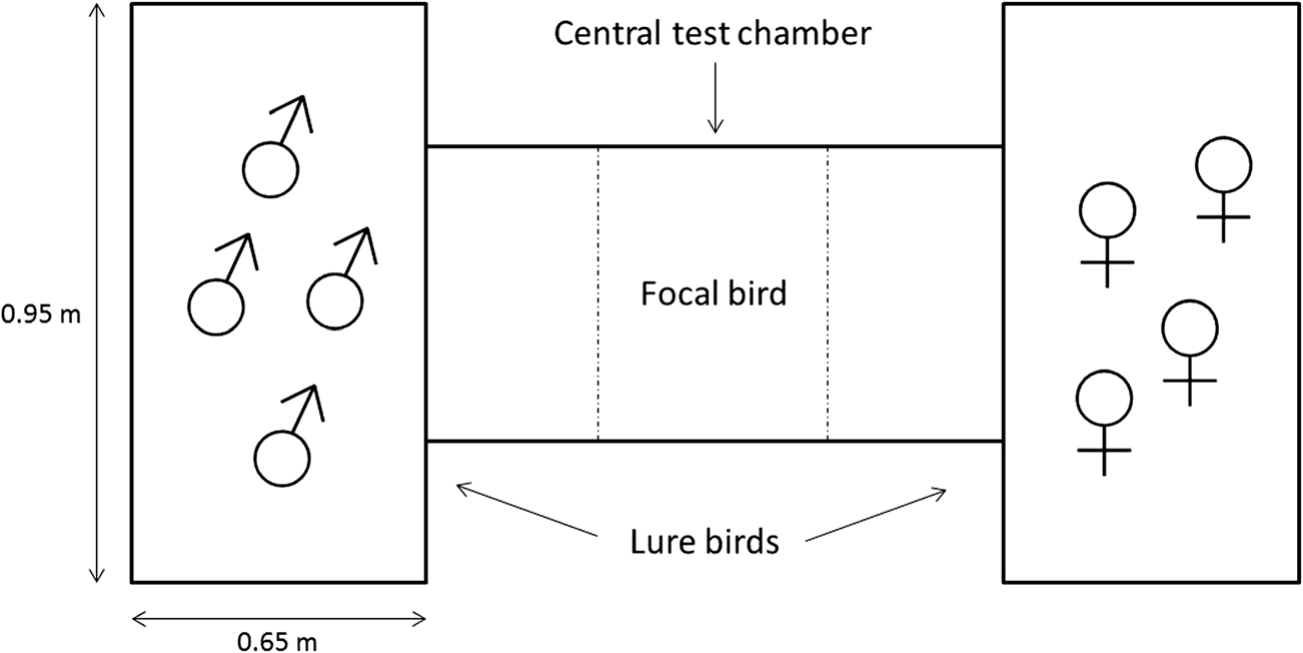
Layout of the social preference test

In 2010, we exposed 134 males and 134 females to binary choice tests during a period when they were sexually dimorphic (3–5 weeks old), and in 2016, we exposed an additional 10 males and 10 females aged 6 days when the pheasants are sexually monomorphic to the same choice test. The apparatus (Fig. 1) consisted of three chambers (2010, 0.95 m × 0.65 m; 2016, 0.23 m × 0.46 m): a central testing chamber and two end chambers separated by transparent plastic mesh. The central test chamber was divided into three equally sized areas (2010, 0.32 m × 0.65 m; 2016, 0.15 m × 0.23 m). In one of the end chambers, we placed a group of males (2010, *n* = 4; 2016, *n* = 3) that were unfamiliar to the focal individual, and in the opposite chamber, we placed the same number of unfamiliar females. The side of the sex was counterbalanced to control for side bias. These audience birds were regularly changed to reduce any potential confounds of stress that may result from being isolated from their familiar housing. A test bird was placed into the central test chamber. The individual was allowed a habituation period (2010, 5 min; 2016, 3 min), followed by a continuous focal follow for 5 min. We recorded the time the focal bird spent in each third of the test chamber, with time spent in the third next to the audience of one sex indicating a preference for that sex. We separated the birds into two distinct groups based on size dimorphism. The first group consisted of all birds tested in 2010, as these birds were all sexually dimorphic, being 3–5weeks old, when they entered the testing chamber. The second group consisted of all birds tested in 2016, which were sexually monomorphic in body size when they entered the testing chamber, being <1 week old. We used a generalised linear model with normal distribution to test the sex differences between the times (arcsine-transformed) a focal individual spent with the same or opposite sex (Table 3).

### Statistical analysis

We conducted all analyses using R statistical software (R Development Core Team 2014) using lme4 package (Bates et al. 2014). When necessary, we controlled for over-dispersion in our data by including an observational level random effect which allows scale parameters to be correctly modelled to validate a Poisson distribution (Elston et al. 2001). Variables considered in each GLMM and data distributions are summarised in Table 3. In all models, we simplified the maximum model using a backward stepwise deletion of non-significant terms based on likelihood ratios, until the removal of variables increased the model deviance, the minimum model. All models were visually inspected for sphericity, homogeneity of variance, normality of error and linearity where required. It was not possible to collect data blind to the sex of the focal individual when they were >~4 weeks old, due to the birds being conspicuously dimorphic in their plumage. Prior to this, focal birds were identified by their numbered tags and subsequently sexed at release. No bird was used more than once in any of the behavioural observations or behavioural tests. For all values, we provide the mean ± 1 standard error of that mean.

## Results

### Do pheasants show a preference to associate with their own sex within a semi-natural environment, and does this change with age?

**Fig. 2:**
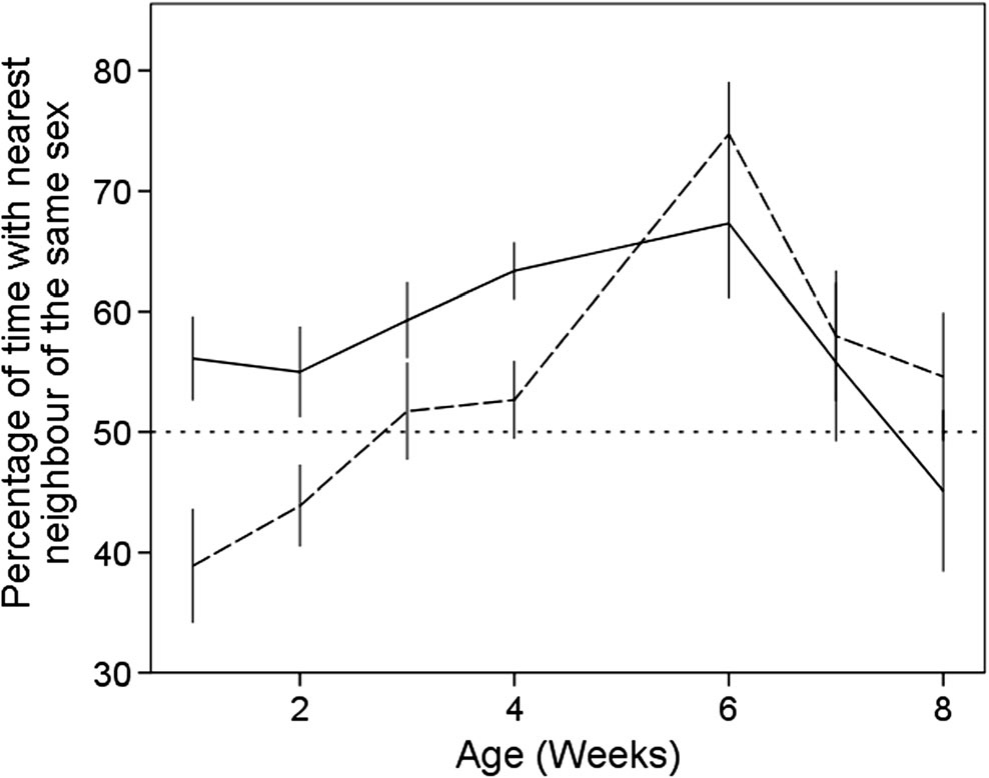
The mean percentage of time that male (*dashed line*) and female (*solid line*) pheasants spent with their own sex during focal observation during the entire study. The *dotted line* represents random assortment. *Error bars* indicate ±1 SE

When 1 week old, females associated more with their own sex, whereas males exhibited random assortment. Preference to be associated with their own sex differed with the sex of the bird and its age. Females consistently preferred to associate with their own sex during the first 6 weeks, and then exhibited more random assortment in weeks 7 and 8. Males increased their preference for males in the first 6 weeks, and then also reverted to a more random assortment in weeks 7 and 8 (Sex*Age*Nearest Neighbour, estimate  = 1.41, SE = 0.32, *Χ* ^2^ = 19.87, *P* < 0.001, Fig. 2).

### When do sexes differ in body size during development?

**Fig. 3:**
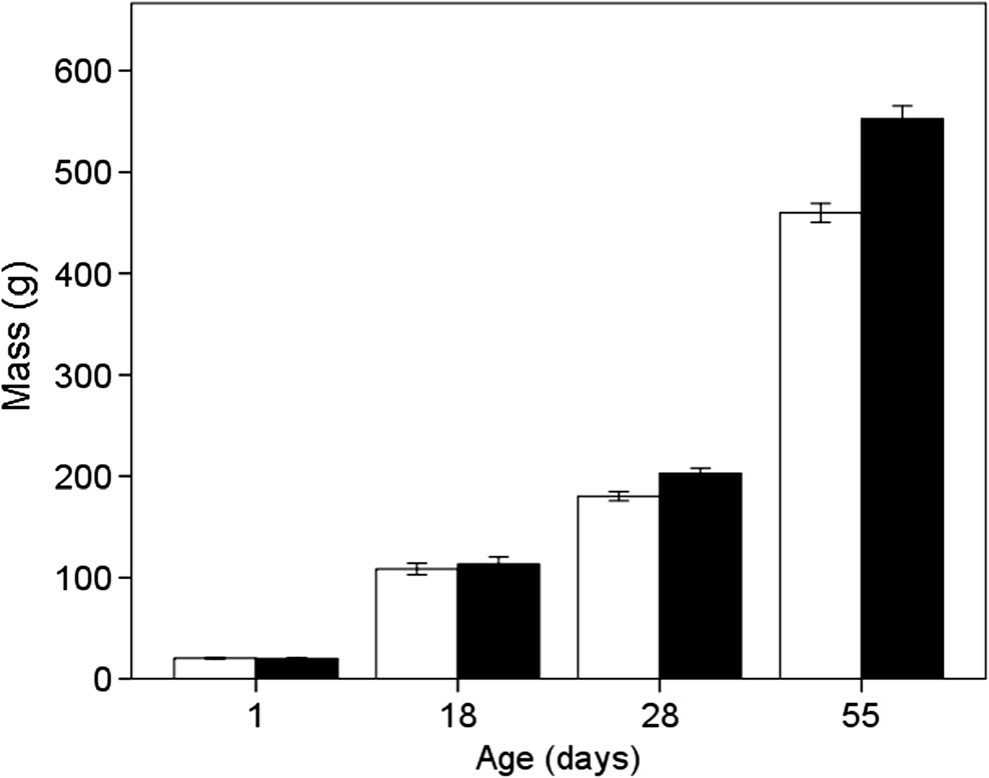
The mean mass of male (*black bars*) and female (*white bars*) pheasants weighed at day 1, day 18, day 28 and day 55. *Error bars* indicate 95% CI

When analysing a subset of birds, we found that there was no sex differences in mass for birds at 1 day old (Sex, estimate = −0.35, SE = 0.49, *Χ* ^2^ = 0.51, *P* = 0.47) or at 18 days old (Sex, estimate = 4.8, SE = 4.42, *Χ* ^2^ = 1.21, *P* = 0.27). However, birds were sexually dimorphic in body mass by day 28 (Sex, estimate = 36.26, SE = 10.57, *Χ* ^2^ = 41.09, *P* < 0.001), and this increased by day 55 (Sex*Age, estimate = 2.06, SE = 0.21; *Χ* ^2^ = 80.51, *P* < 0.001) (Fig. 3).

### Is segregation due to differences in diet selection?

Males (6.68 ± 0.23) did not differ from females (6.12 ± 0.23) in the number of different food types consumed when presented with 10 choices (Sex, estimate  = 0.088, SE = 0.07, *Χ* ^2^ = 1.79, *P* = 0.18). The percentage of the female population that chose a certain food item in the first three choices did not differ from that of the male population (*χ* ^2^_c_ = 13.37, *P* = 0.15).

### Do sexes differ in the time spent foraging or resting?

Regardless of whether an individual was observed during a period when they were sexually dimorphic or monomorphic in body size, males did not differ from females in their likelihood of performing foraging and resting behaviours (Table 4). However, individuals observed when dimorphic in body size (0.16 ± 0.02) had a lower likelihood of performing foraging behaviours compared to individuals observed when monomorphic in body size (0.22 ± 0.02) (Table 4). Individuals observed when monomorphic in body size (0.16 ± 0.02) did not differ in the likelihood of performing resting behaviours compared to individuals observed when dimorphic in body size (0.16 ± 0.03) (Table 4).

**Table 4:** Summary statistics of binomial GLMMs testing for predictors of foraging and resting behaviour

Behaviour	Variable	Estimate	SEM	*Χ* ^2^	*P*
Foraging					
Full model	Sex × degree of dimorphism	0.39	0.20	3.71	0.05
	Sex	−0.16	0.10	2.42	0.12
Minimum model	*Degree of dimorphism*	*−0.31*	*0.10*	*9.02*	*0.003*
Resting					
Full model	Sex × degree of dimorphism	0.2	0.22	1.43	0.23
	Degree of dimorphism	−0.02	0.11	0.03	0.86
	Sex	0.19	0.11	2.95	0.09

Significant terms and values are shown in italic type

### Does foraging efficiency differ between sexes?

Males (55.89 s ± 11.27) were more than twice as fast as females (133.13 s ± 17.39) to eat a tethered cricket after detection (*W*_117_ = 2372.5, *P* = 0.001). Males (99.87 s ± 13.80) were also ~40% faster than females (139.48 s ± 16.04) to catch the roboworm (*W*_186_ = 5118.5, *P* = 0.018).

### Does aggression differ between the sexes?

**Fig. 4:**
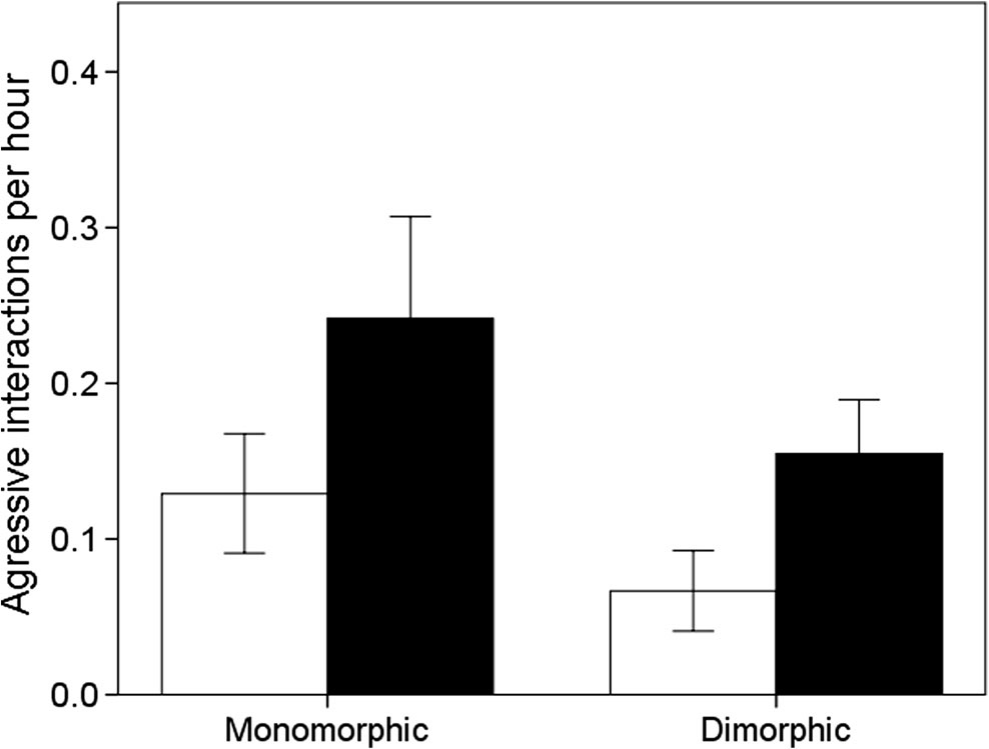
The mean amount of aggression shown by male (*black bars*) and female (*white bars*) pheasants during periods when pheasants are monomorphic (1–3 weeks old) and dimorphic (4–6 weeks old) in body size. *Error bars* indicate ±1 SE

**Fig. 5:**
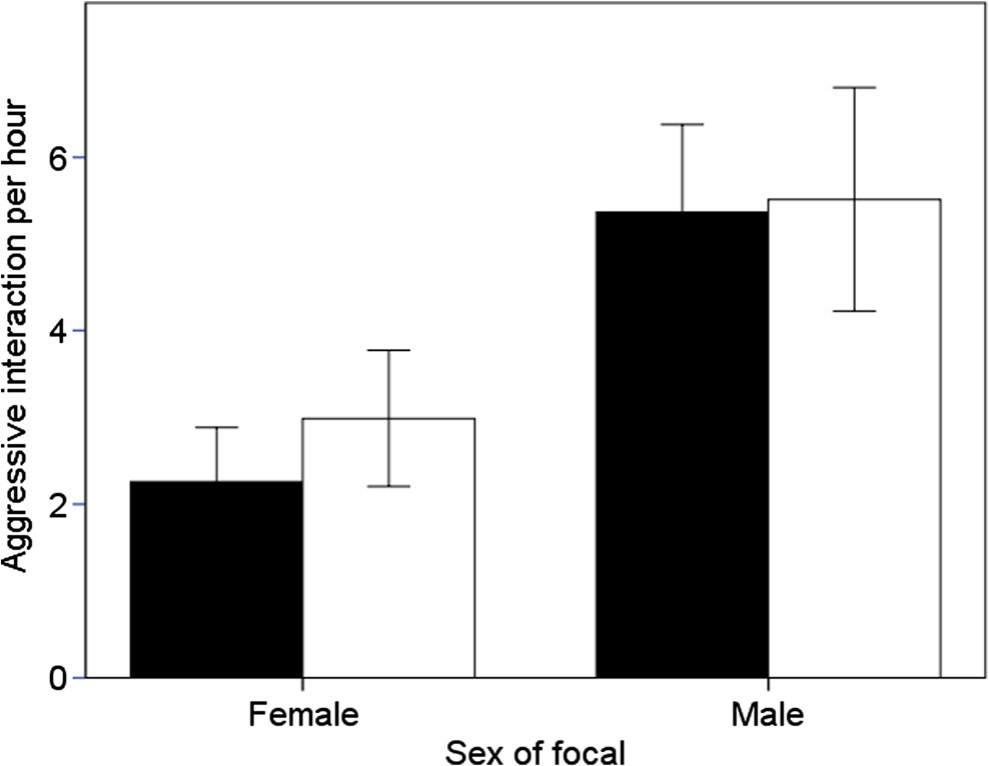
The mean amount of aggression directed from a focal individual (male or female) towards a male (*black bar*) or female (*white bar*) pheasant per hour. *Error bars* indicate ±1 SE

Birds of both sexes were more aggressive during periods when monomorphic in body size than when they were dimorphic (Table 5; Fig. 4). Males were more aggressive than females (Table 5; Fig. 5). A focal individual, regardless of sex or age of testing, did not differ in which sex they directed their aggressive interaction towards (Table 5; Fig. 5).

**Table 5:** Summary statistics of repeated measures of GLMM for aggression, looking at which sex an individual prefers to be aggressive towards (opposite sex or same sex), with the sex and age of the focal individual as predictors

Variable	Estimate	SEM	*Χ* ^2^	*P*
Full model				
Sex × degree of dimorphism × direction	−3.7	0.72	0.26	0.61
Degree of dimorphism × direction	−0.54	0.35	2.43	0.12
Sex × direction	0.28	0.37	0.60	0.44
Sex × degree of dimorphism	−0.43	0.63	0.45	0.50
Direction	−0.09	0.17	0.29	0.59
Minimum model				
*Sex*	*0.86*	*0.31*	*7.48*	*0.0062*
*Degree of dimorphism*	*0.67*	*0.18*	*4.66*	*0.03*

Both the full model and the minimum model are shown. Significant terms and values are shown in italic type

### Could segregation be due to active preference?

**Fig. 6:**
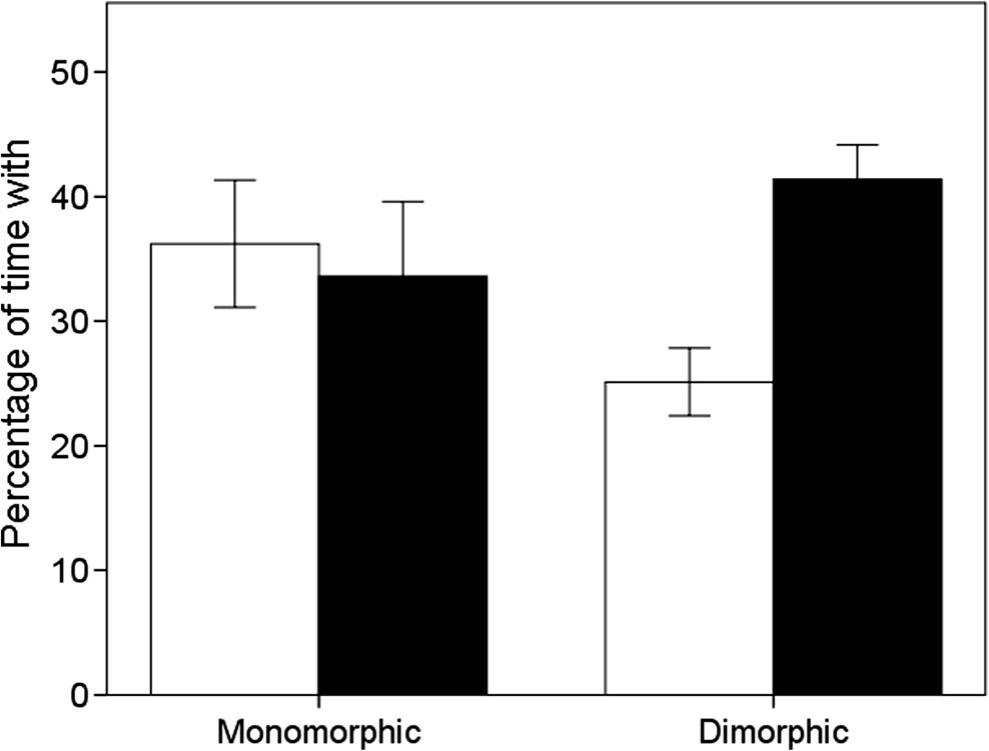
The mean percentage of time a focal individual spent with their own sex (*black bar*) and their opposite sex (*white bar*) when tested in isolation when sexually monomorphic (aged 1–3 weeks) and when sexually dimorphic (aged 4–6 weeks) in body size. *Error bars* indicate ±1 SE

When tested in isolation, both males and females preferred to spend time with their own sex when sexually dimorphic in body size; however, neither sex showed a preference for a particular sex during tests when monomorphic in body size (Table 6; Fig. 6).

**Table 6:** Summary statistics of repeated measures of GLMM, testing for the preferences of a focal individual towards their own or opposite sex when given a binary choice, with the sex and state of sexual dimorphism (monomorphic or dimorphic in body size) of the focal individual as predictors

Variable	Estimate	SE	*Χ* ^2^	*P*
Full model				
Preference × sex × degree of dimorphism	0.14	0.28	0.26	0.61
Sex × degree of dimorphism	−0.04	0.14	0.08	0.77
Preference × sex	−0.13	0.09	1.84	0.18
Sex	0.01	0.05	0.04	0.85
Minimum model				
*Preference × degree of dimorphism*	*−0.29*	*0.14*	*4.30*	*0.04*
Degree of dimorphism	0.18	0.10	0.32	0.57
*Preference*	*0.25*	*0.05*	*18.97*	*<0.001*

Both the full model and the minimum model are shown. Significant terms and values are shown in italic type

## Discussion

Pheasants reared in the absence of parental influence from 1 day old in a controlled environment, with equal habitat availability and food provision and with no predator risk, showed clear preferences to associate with their own sex in the first weeks of life.

Although they are sexually dimorphic in body size by 3 weeks old, we found no evidence that this influenced sexual differences in activity budgets or dietary preference. Instead, social preferences, clearly seen in binary choice tests conducted after the onset of sexual dimorphism, could drive the patterns of association. These social preferences could have manifested because males are more aggressive than females, possibly in efforts to assert dominance or increase future reproductive success.

Females showed a preference to associate with other females within a semi-natural environment in the first week of life, whereas males initially exhibited random associations. As birds got older, the preferences for their own sex increased for both sexes. Sexual segregation during early development is rarely reported outside of humans, and in those species where it has been reported, it is typically much later in life; juvenile cowbirds have been shown to aggregate as juveniles (less than 75 days old) (Kohn et al. 2011), and spider monkeys segregate prior to adulthood (prior to 50 months old) (Rodrigues 2014). However, possible causes for why this occurs were not formally tested. The random assortment that we observed at 7 and 8 weeks was likely due to the relatively small size of the housing system, constraining the expression of preferential assortment during the final weeks of the study, with individuals being forced to associate with others because of space constrictions. We believe that it is the dimensions of the housing that caused the pheasant to assort randomly, rather than returning to a previous preference that was observed in the first few weeks, because pheasants released into larger pens at the 10 weeks showed clear segregation (MAW et al. unpubl. data).

Pheasants showed no detectable differences in body mass on hatching or in their first 18 days of life; however, by 28 days, males were 1.12 times larger than females, and at 55 days, males were 1.20 times larger than the females. There is a lack of information on size dimorphism in birds that sexually segregate, but these are similar levels to sexual dimorphism reported in adult red deer (1.33; Clutton-Brock et al. 1982; Weckerly 1998), although slightly less than merino sheep (1.50; Michelena et al. 2004) and big horn sheep (1.43; Blood et al. 1970). Consequently, hypotheses based on sexual dimorphism could be expected to explain segregation even in juvenile pheasants, as well as those hypotheses which look at non-sexually dimorphic characteristics.

Rearing pheasants in a predation-free environment, with equal habitat availability prior to maturation, nullified the effects of the Predation Risk Hypothesis. Likewise, rearing with equal habitat availability combined with a homogenous diet of commercial pellet excluded the Forage Selection Hypothesis. Body size dimorphism did not influence the dietary preference of birds that had been reared with access to a diverse diet and presented with a dietary preference test during the period when they were sexually dimorphic in body size, again failing to support the Forage Selection Hypothesis.

Neither time spent foraging nor time spent resting differed between the sexes during periods when the birds were monomorphic or dimorphic in body size. This is surprising because differences in foraging behaviour have been suggested to explain why some ungulates sexually segregate (Ruckstuhl 1998). One explanation why male pheasants may not need to spend more time foraging than females, despite the larger size, is that they are more efficient at acquiring their food. In support of this, we found that males were indeed able to capture a live, or simulated live, novel prey item faster than females. The lack of differences in resting behaviour is also surprising as body size dimorphism corresponds to differences in gut morphologies which, in ungulates, can result in differential time spent resting, digesting and ruminating (Ruckstuhl and Neuhaus 2002). The pheasant ceca is analogous to the hind guts of ungulates, but chicks, even when sexually dimorphic in body size, did not differ in their time spent resting. One reason for this lack of difference in resting behaviour may be that the sexual body size dimorphism did not correspond to a difference in ceca size between the sexes, or the difference was not large enough to drive behavioural differences. We may find that the provision of chick crumb, ad lib and in excess, could mean that dietary requirements are met easily, and therefore, we would not expect to see any difference in time allocated to foraging and resting between the sexes. With provision of scarcer or lower-quality forage, time allocated to foraging or resting may differ between the sexes. Interestingly, pheasants that were observed during a period when they were monomorphic in body size foraged longer than those observed when dimorphic in body size. Such differences is not attributed to spending more time resting, as this did not differ with body size dimorphism; however, it could be attributed to greater time spent conducting behaviours not measured in this study, such as vigilance. Difference in time spent foraging could, however, be a consequence of improved foraging and handling skills. With no differences in resting or foraging behaviours between the sexes, we can suggest that neither the Activity Budget Hypothesis nor the Energetic/Behavioural Synchrony Hypothesis explains sexual segregation in young pheasants.

Instead, patterns of preferential assortment appeared early in life. When presented with a binary choice during the first weeks of life when pheasants are sexually monomorphic, both sexes showed no preferences for either sex. However, 3 to 5 weeks later, when pheasants are dimorphic in body size, both sexes preferred to spend time with their own sex. This indicates that active preferences could drive the patterns of segregation observed in our semi-natural conditions. One explanation for why such preferences were expressed early in life is that male pheasant chicks were more aggressive than females.

Males being more aggressive than females is a feature that is common in species which sexually segregate (e.g. mouflon sheep (Guilhem et al. 2006), and humans (Pellegrini 2004)). Aggressive interactions in chicks are sometimes considered analogous to mammalian play behaviour (Mench 1988); however, these interactions can cause severe harm and death and are the reasons why bits and masks are used in pheasant-rearing facilities to reduce their damaging effects (Butler and Davis 2010). In polygynous ungulates, play behaviour during development may increase their later reproductive success (Clutton-Brock et al. 1982; Weckerly 1998). Pheasants adopt a system of harem defence polygyny, where an adult male obtains a territory through agonistic male-male interactions and subsequently mates with females within that territory (Ridley and Hill 1987). Non-territorial males seldom mate (Mateos 1998). Therefore, increased aggressive male-male interaction during ontogeny may facilitate future territory acquisition and so enhance reproductive success.

When the pheasants were monomorphic in body size, the males did not choose social partners based on their sex; but as the sexes differentiated, males preferred to interact with other males, perhaps in an effort to assist the development of their own fighting skills to enhance their own reproductive success. Sexually dimorphic ungulate males benefit from experiencing conflict with other males, rather than smaller females, in order to develop useful fighting skills (Clutton-Brock et al. 1982). Male mouflon sheep actively seek other males during ontogeny (Le Pendu et al. 2000). However, during the period when pheasants were dimorphic in body size, we did not see males being more aggressive to other males, even though they preferentially associated with them. Perhaps the artificially close proximity of all birds in captivity provoked high levels of aggression towards all birds, and so masked sex-specific patterns of aggression.

For females, the motivation may be different; associating with other females avoids the male pseudo-sexual and agonistic behaviours (LaGory et al. 1991). When in mixed-sex groups, we found that females would assort with their own sex even prior to size dimorphism, suggesting that they were avoiding males even in the first week of life, although this was not mirrored in the binary tests conducted in their first week. We suspect that females are actively avoiding aggression from males as it happens in mixed-sex groups, but have not yet developed a sufficiently strong preference or discriminatory ability to cause them to avoid males when tested in isolation in their first week. Avoidance of harassment and injury by aggressive males is not uncommon. Human infant females will avoid areas that host rough and vigorous male behaviours (Harper and Sanders 1975; Pellegrini 1992). Whether female pheasants were actively avoiding males or preferentially seeking out females cannot be teased apart from this study.

Studies that investigate competing hypotheses to explain segregation often make efforts to eliminate all but one hypothesis as a likely explanation (Ruckstuhl and Neuhaus 2002; Bowyer and Kie 2004). This becomes difficult when assessing individuals in the wild due to lack of environmental controls, ignorance of early life circumstances and inability to conduct experiments. As such, many studies conclude that observed segregation is based upon multiple hypotheses working in concert, sequentially or in any combination, and frequently fail to (be able to) consider early life developmental factors (Loe et al. 2006; Bonenfant et al. 2007; Ruckstuhl 2007; Alves et al. 2013). This may be confusing our understanding of how and why animals segregate. Firstly, ontogenetic factors could have a greater impact on sexual segregation than previously thought. Although ontogenetic behaviour and subsequent preferences are believed to impact aspects of ungulate sexual segregation (Michelena et al. 2004; Pérez-Barbería et al. 2005), these factors are rarely studied. Additionally, although many studies look at segregation by adults in the wild, it may be that testing between hypotheses when segregation has already occurred risks ignoring the underlying influence of early social factors (Guilhem et al. 2006), and therefore overemphasises the importance of hypotheses related to sexual dimorphism. Secondly, current studies of sexual segregation focus on taxa with highly altricial young and small generation sizes, such as ungulates (Bowyer 2004; Ruckstuhl and Neuhaus 2002). This means that there are few opportunities for interactions during ontogeny (Ruckstuhl 1999), and therefore, in these systems, early life effects may be reduced. However, in systems that allow for large aggregations of young, e.g. in birds with large clutch size or in fish, we may find that segregation may have evolved according to different selective forces, with early life social factors having more influence. The pheasant provides a new study system which, when reared in captivity, can overcome some of these problems, and therefore offers new insights into the development and expression of sexual segregation.
